# Intravitreal Delivery of Melatonin Is Protective Against the Photoreceptor Loss in Mice: A Potential Therapeutic Strategy for Degenerative Retinopathy

**DOI:** 10.3389/fphar.2019.01633

**Published:** 2020-02-12

**Authors:** Chong Li, Yi Tian, Anhui Yao, Xiaobing Zha, Jianbin Zhang, Ye Tao

**Affiliations:** ^1^Department of Neurosurgery, PLA General Hospital, Beijing, China; ^2^Department of Oncology, Xijing Hospital, Fourth Military Medical University, Xi'an, China; ^3^Department of Rehabilitation, Zhongshan Hospital, Fudan University, Shanghai, China; ^4^Department of Occupational and Environmental Health, Ministry of Education Key Lab of Hazard Assessment and Control in Special Operational Environment, School of Public Health, Fourth Military Medical University, Xi'an, China; ^5^Department of Ophthalmology, Henan Provincial People’s Hospital, Zhengzhou University, People’s Hospital, Zhengzhou, China

**Keywords:** neuroprotection, degeneration, retina, toxicity, therapeutics

## Abstract

Melatonin is a circadian hormone with potent cytoprotective effects. Retinitis pigmentosa (RP) comprises a heterogeneous group of inherent retinopathies that characterized by the photoreceptor death in bilateral eyes. The N-methyl-N-nitrosourea (MNU) administered mouse is a type of chemically induced RP model with rapid progressive rate. We intend to study the melatonin mediated effects on the MNU administered mice. Melatonin was delivered into the vitreous body of the MNU administered mice. Subsequently, the melatonin treated mice were subjected to histological analysis, optokinetic behavior tests, spectral-domain optical coherence tomography (SD-OCT), and electroretinogram (ERG) examination. Multi-electrodes array (MEA) was used to analyze the status of visual signal transmission within retinal circuits. Biochemical analysis was performed to quantify the expression levels of antioxidative enzymes, oxidative stress markers, and apoptotic factors in the retinas. The intravitreal injection of melatonin ameliorated effectively the MNU induced photoreceptor degeneration. Melatonin therapy mitigated the spontaneous firing response, and preserved the basic configurations of visual signal pathway in MNU administered mice. MEA is effective to evaluate the pharmacological effects on retina. Of note, the cone photoreceptors in degenerative retinas were rescued efficiently by melatonin therapy. Melatonin afforded these protective effects by modulating the apoptotic cascades and alleviating the oxidative stress. These findings suggest that melatonin could act as an alternative treatment for degenerative retinopathy. Melatonin might be used in combination with other therapeutic approaches to alleviate the photoreceptor loss and preserve the visual function of RP patients.

## Introduction

Retina is a light-sensitive tissue that located at the posterior pole of eyeball. It is organized into lamellar structures with complex microcircuits that work synergistically to process visual signal. Insults to the retina would cause irreversible visual impairments (Briggman et al., 2011; Galli-Resta et al., 2018). Retinal pigmentosa (RP) is a collection of inherited retinopathies that characterized by the progressive photoreceptor death. The pathological process of RP is influenced by a spectrum of molecular, cellular, and tissue-level factors (Lemos Reis et al., 2015). In view of the enormous heterogeneity implied in etiology, RP patients always have highly variable onset points, progressive dynamics, and prognosis outcomes (Cai et al., 2014; Falsini et al., 2016). These variations make the accurate diagnosis extremely challenging. In clinical settings, no medication can halt the photoreceptor death and visual devastations in RP patients (Khan et al., 2017; Rapino et al., 2018). Accumulating evidences suggest that the oxidative stress contributes to the photoreceptor apoptosis of RP. Excessive oxidative radicals would perturb redox metabolism, alter mitochondrial membrane permeability, and induce cytochrome c leakage in photoreceptors (Mao et al., 2014; Hoffman et al., 2015). As long as the surplus oxidative radicals are scavenged by deoxidizer, photoreceptors might survive longer and function well under favorable circumstances. This notion is further corroborated by the fact that molecules with antioxidative potency can improve the visual function of RP patients (Crooke et al., 2012; Koushan et al., 2013; Narayan et al., 2016).

Photoreceptors are extremely vulnerable to oxidative insults. Their homeostasis necessitates abundant antioxidants that directed against the reactive radicals (Nowak, 2013). Melatonin is an indoleamine which is synthesized mainly by the pineal gland in a circadian fashion. Retina is a primary recipient of circadian signals and is considered as a “light sensitive ocular clock” (Flynn-Evans et al., 2014; Besharse and McMahon, 2016). In particular, melatonin is also synthesized by the photoreceptors to improve visual sensitivity (Gianesini et al., 2016; Hull et al., 2018). As the melatonin receptors are intensively localized at the synapse terminals of photoreceptors, it is highly possible that melatonin might mediate beneficial effects on photoreceptors (Owino et al., 2018). Sever lines of evidences suggest that exogenous melatonin confers cytoprotective effects on the retina (Tosini et al., 2012). Melatonin can act synergistically with vitamin E to ameliorate the nitric oxide-induced lipid peroxidation in retina (Siu et al., 1999). Comparison analysis shows that melatonin is approximately 100 times more potent in inhibiting the light-induced oxidative impairments than does the vitamin E (Marchiafava and Longoni, 1999). Melatonin can also mitigate the oxidative stress and prevent the abnormal vascular congestion in diabetic retinas (Salido et al., 2013). Another *in vitro* study shows that exogenous melatonin promotes the survival of rod photoreceptors and retinal pigment epithelial cells, both of which are implicated in the RP pathogenesis (Liang et al., 2004). Moreover, exogenous melatonin is also protective against ocular disease models, such as the glaucomatous optic neuropathy, retinal ischemia-reperfusion injury, and retinopathy of prematurity (Siu et al., 2006). Melatonin exerts these protective actions by scavenging the oxygen free radicals, stimulating the activity of cellular antioxidative enzymes, stabilizing the mitochondrial electron transport chain, and modulating the expression of apoptotic genes (Blasiak et al., 2016).

N-methyl-N-nitrosourea (MNU) is an alkylating toxicant that induces rapid photoreceptor cell death *via* systemic administration (Tsubura et al., 2011). The MNU administered mouse is typically used as a chemically induced RP model (Tsuruma et al., 2012). MNU interacts with DNA and yields the 7-medGua DNA adduct selectively in photoreceptor nuclei at 6 h after MNU administration. The apoptosis cascade in photoreceptors is activated at 12 h after MNU administration as evidenced by the down-regulated Bcl-2 level. At this time point, internucleosomal DNA fragmentation is seen in the photoreceptors (Tsubura et al., 2010). At 24 h after MNU administration, the first evidence of histological alterations can be detected. Photoreceptors show pyknosis of the nuclei, and shortening of the inner and outer segments (Nakajima et al., 1996a; Nakajima et al., 1996b). At 48 h after MNU administration, the destruction of photoreceptor nuclei is most prominent. Eventually at day 7, active signs of photoreceptor degeneration are indistinct due to photoreceptor loss (Yoshizawa et al., 1999; Yoshizawa et al., 2000; Tsubura et al., 2010). This study is designed to explore the melatonin induced protective effects on photoreceptor degeneration. Melatonin is delivered into the vitreous body of the MNU administered mouse. We aimed to find whether melatonin exerts beneficial effects on the photoreceptor survival, visual function, and visual signal transmission of MNU administered mice. In particular, we intend to quantify the therapeutic efficiency of melatonin *via* topographic analysis. These findings would enrich our understandings of melatonin, and shed light on the development of a new medication for RP.

## Materials and Methods

### Animals and Study Design

**Figure 1:**
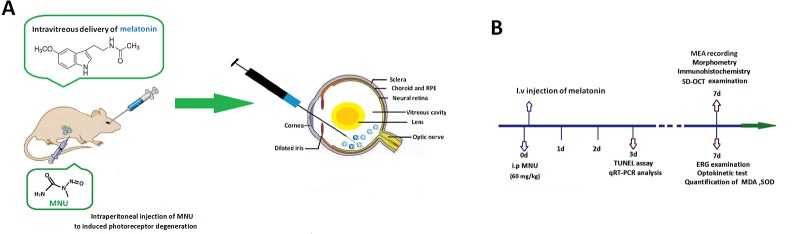
**(A)** Massive photoreceptor degeneration was induced by an intraperitoneal administration of N-methyl-N-nitrosourea (MNU). Melatonin was injected into the vitreous body of MNU administered mice in the therapeutic section. **(B)** A schematic illustration of the experimental protocols.

The animals were handled following the Association for Research in Vision and Ophthalmology (ARVO) guidelines for the Use of Animals in Ophthalmic and Vision Research. All the procedures and protocols were conducted as approved by the Institutional Animal Care and Use Committee of Chinese PLA general hospital (OOC-20187813). Totally 280 mice (C57/BL, 8–9 weeks old with both sexes, body weight range between 19 and 23 g) were used in this study. Animals were maintained in the specific pathogen free facility (18–23°C, 40–65% humidity, 12-h dark/light cycle) with food and water available. These mice were randomly assigned into four subgroups: 1) normal controls: mouse without any pharmacological administration; 2) MNU group: mouse received an intraperitoneal injection of MNU (60 mg/kg; Sigma-Aldrich Corp., MO, USA); 3) MNU+melatonin group: mouse received an intravitreal injection of melatonin (150 μg/kg body weight; Sigma-Aldrich Corp., MO, USA) 2 h post-MNU administration. 4) MNU+vehicle group: mouse received an intravitreal injection of 2 μl vehicle 2 h post-MNU administration. In the dose effects analysis, the MNU administered mouse received an intravitreal injection of melatonin at the dose of 50, 100, 200, and 250 μg/kg, respectively. MNU (Sigma; St. Louis, MO) was kept at −4°C in dark. MNU was dissolved in the physiologic saline containing 0.05% acetic acid just before use. Generally, the MNU induced retinal degeneration accomplishes within 7 days with the dose of 60 mg/kg (Gao et al., 2010; Tsubura et al., 2011). This administered dose has been used in multiple ophthalmological studies (Tsubura et al., 2010). It costs a period of time for experimental animals to recover from trauma after the MNU administration. To minimize their sufferings, we left the mice in shielded cages for 2 h and verify if there was any abnormal symptom in them. If no adverse effect was evident in the MNU administrated animal, the intravitreal injection was then performed. The preparation of melatonin solution followed a previous described method (Andrés-Guerrero et al., 2009; Berger et al., 2017). The melatonin was firstly dissolved in 5% dimethyl sulfoxide (DMSO) and then further diluted with phosphate-buffered saline (PBS) at various concentrations. Control animals received vehicle injection containing the same amount of PBS and DMSO as given to the melatonin treated groups. The dose of melatonin selected in this study was based on data from other investigators who have studied its protective effects against retinopathy (Yilmaz et al., 2004; García-Caballero et al., 2018). **Figure 1** is a schematic illustration of the experiment protocols.

### Optokinetic Behavioral Test

Optokinetic behavior was evaluated *via* a two-alternative forced choice paradigm as described previously (McGill et al., 2012). The response threshold was determined by the stepwise functions of correct track responses. The initial stimulus was set as 0.200 cycle/degree sinusoidal pattern with a fixed 100% contrast.

### Electroretinogram Examination

All the animals were dark adapted for at least 12 h. Subsequently, the ERGs of the mice were recorded by the RETIport system (Roland Consult, Germany) as described previously (Tao et al., 2015).

### Spectral-Domain Optical Coherence Tomography

Mice were transferred to the recording plane of an ultrahigh-resolution instrument when they were still anesthetized (Bioptigen, Durham, NC, USA). Methylcellulose lubricant (Allergan Inc, Dublin, Ireland) was applied on the corneas of mice, and the probe was positioned near the cornea until the retinal image appeared on the screen. A corresponding box was focused on the optic nerve head (ONH) for orientation and eight measurements at the same distance (0.3 mm) from the edge of the ONH on either side were executed.

### Multi-Electrode Array Recording

Multi electrode array (MEA) recording was performed following a previously described method (Tao et al., 2015). Briefly, retinal specimens were placed in the recording chamber of the electrodes array. The analog extracellular responses of retinal neurons were recorded by the MED-64 system (Alpha Med Sciences, Osaka, Japan). The waveforms of field potentials were processed with a band pass filter (100 to 3,000 Hz) for spike evaluation. Peristimulus time histograms (PSTHs) and the raster plots were used for retinal ganglion cells (RGCs) categorization. ON and OFF responses were analyzed according to the PSTHs.

### Histological and Immunohistochemical Analysis

Retinal sections and whole mount preparations were prepared following a previously described method (Tao et al., 2015). For immunohistochemistry, the peanut agglutinin (PNA) conjugated to a Alexa Fluor 488 (1:200, Invitrogen, USA), S-cone opsin, or M-cone opsin antibodies (1:400, Millipore, MA, USA) were incubated with retinal specimen, respectively. After thorough rinses with PBS, the retinal specimens were incubated in Cy3-conjugated anti-rabbit immunoglobulin G (IgG) (1:400, Jackson ImmunoResearch Laboratories, USA) and 4′,6-diamidino-2-phenylindole (DAPI). Cone cells within four 420x420 μm bins surrounding the ONH were quantified using AxioVision Rel. software.

### Terminal Deoxyuridine Triphosphate Nick-End Labeling Assay

Terminal deoxyuridine triphosphate nick-end labeling (TUNEL) assay was conducted using the *in situ* cell death detection POD Kit (Roche Diagnostics GmbH, Mannheim Germany). Apoptotic index (AI) of the outer nuclear layer (ONL) was calculated as (number of TUNEL-positive nuclei/total number of photoreceptor cell nucleix100).

### Quantitative Reverse Transcription-Polymerase Chain Reaction

Mice were killed and their eyes were enucleated. Total RNA was extracted from retinal patches with a commercial reagent (TRIzol, Gibco Inc., Grand Island, NY), followed by complementary DNA (cDNA) synthesis using the μMACS™ DNA Synthesis kit (Miltenyi Biotec GmbH, Bergisch-Gladbach, Germany). The primers used in quantitative real-time (qRT)-PCR were: Bax: 5'-AGCTCTGAACAGATCATGAAGACA-3' (forward) and 5'-CTCCATGTTGTTGTCCAGTTCATC3' (reverse); Bcl-2:5'-GGACA ACATCGC TCTGTG GATGA-3' (forward) and 5'-CAGAGACAGCCAGGAGAAATCAA-3' (reverse); caspase-3: 5'-TGTCGATGCAGCTAACC-3' (forward) and 5'-GGCCTCCACT GGTATCTTCTG-30 (reverse); Calpain-2: 5'-CCCCAGTTCATTATTGGA GG-3' (forward) and 5'-GCCAGGATTTCCTCATTCAA-3' (reverse). All primers were quality controlled by sequencing the template on a genetic ABI analyzer (Applied Biosystems Inc., Foster City, CA, USA). The results were normalized with housekeeping gene beta-actin. Reactions were performed with SYBRR Green Master Mix (Bio-Rad Laboratories, Reinach, Switzerland) on a real-time CFX96 Touch PCR detection system (Bio-Rad Laboratories, Reinach, Switzerland). The amplification program consisted of polymerase activation at 95°C for 5 min and 50 cycles of denaturation at 95°C for 1 min, annealing and extension at 95°C for 30 s. Duplicate RT-qPCR reactions were performed for each gene to minimize individual tube variability, and an average was taken for each time point. Threshold cycle efficiency corrections were calculated, and melting curves were obtained using cDNA for each individual-gene PCR assay. The relative expression levels were normalized and quantified to obtain the ΔΔCT values (DATA assist Software v2.2, Applied Biosystems).

### Determination of Antioxidative Enzymes and Oxidative Stress Marker Levels

Retina tissue was added into the PBS containing 0.5% Triton X-100 (pH 7.4) and then was homogenized in ice cold by Grinders. The tissue was centrifuged at 500 g for 5 min at 4°C. The suspension was assayed for protein contents to normalize enzyme activity and content of oxidative stress markers. Superoxide dismutase (SOD) activity and malondialdehyde (MDA) concentration were measured as described previously (Du et al., 2018). The Cu-Zn-SOD activity was analyzer with the SOD Assay Kit-WST (Jiancheng Biotech Ltd., Nanjing, China). One unit (U) of Cu-Zn-SOD activity was defined as the amount of enzyme causing half inhibition in the nitroblue tetrazolium reduction rate. A spectrophotometer with ultra-micro-cuvettes was used to measure the absorbance values. The absorbance value of each sample in an ultra microcuvette was measured on a spectrophotometer at 550 nm, and the value was expressed as U/mg protein. The concentration of MDA was assessed using a thiobarbituric acid (TBA) colorimetric assay under the guidance of the manufacturer's protocol (Jiancheng Biotech Ltd., Nanjing, China). The intensity of the resulting pink color was read at 532 nm, and the lipid peroxide levels (formed MDA) were expressed as nmol/mg protein. The manganese-dependent SOD (Mn-SOD) activity was measured using commercially available kits under the guidance of the manufacturer's instructions (Jiancheng Biotech Ltd., Nanjing, China). The Mn-SOD activity was expressed as U/mg protein. The 8-hydroxy-2'-deoxyguanosine (8-OHdG) concentration was quantified by a by competitive ELISA assay kit (Jiancheng Biotech Ltd., Nanjing, China) under the guidance of the manufacturer's protocol. The 8-OHdG concentration was expressed as µg/mg DNA.

### Statistical Analysis

Statistical difference was processed using the ANOVA analysis followed by Bonferroni's *post-hoc* analysis. All the values are presented as mean ± standard deviation (SD). *P* value < 0.05 was considered statistically significant.

## Results

### Melatonin Mediated Effects on Photoreceptor Survival

**Figure 2:**
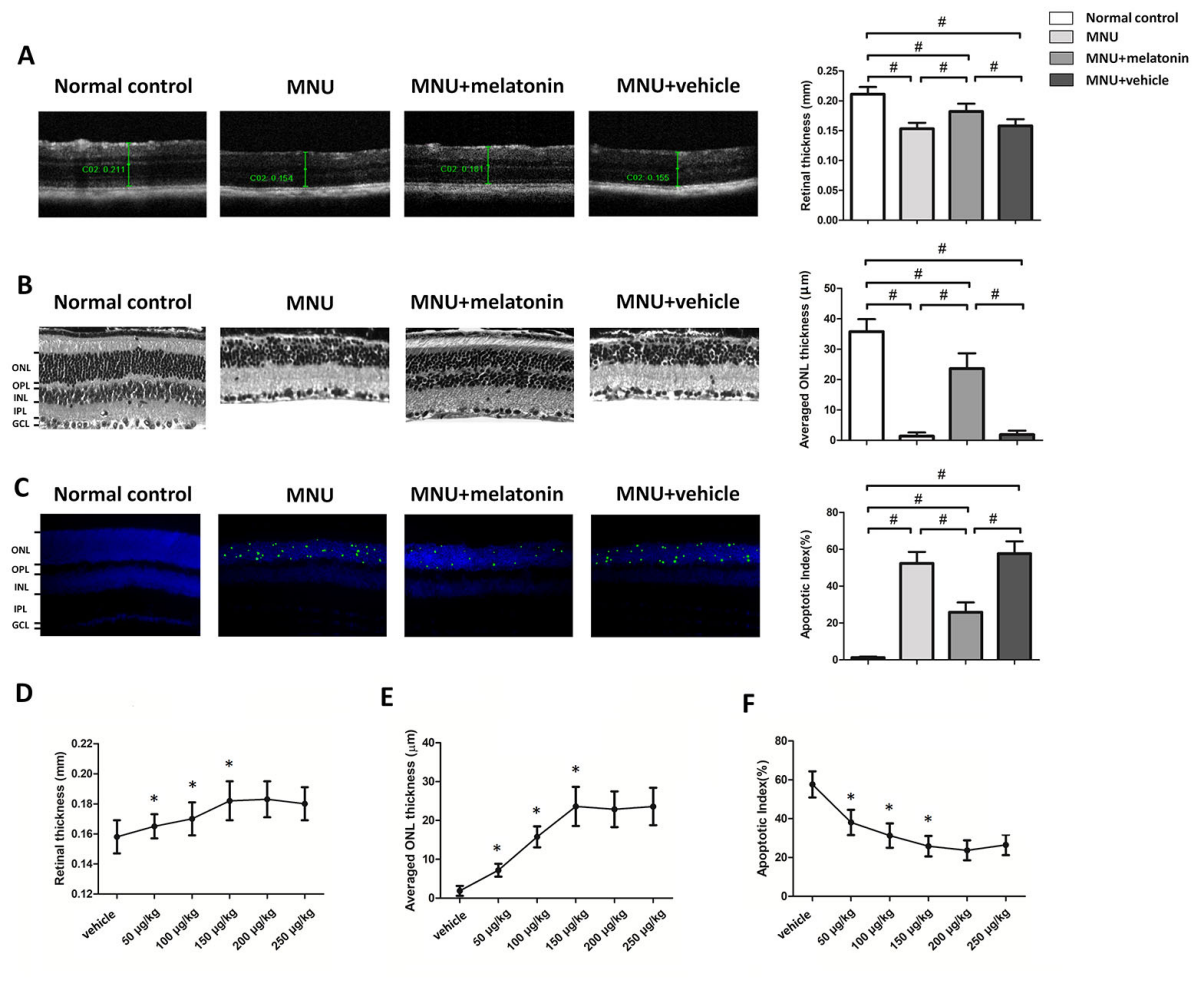
**(A)** Optical coherence tomography (OCT) examination showed clear differences in the retinal thickness among the four animal groups. The retinal thickness of the melatonin treated mice was significantly larger compared with the N-methyl-N-nitrosourea (MNU)+vehicle group. **(B)** The retina structure of the normal controls was highly organized, whereas the retinal structure of MNU group was severely destroyed. The average ONL thickness of the melatonin treated mice was significantly larger compared to MNU group. **(C)** The terminal deoxyuridine triphosphate nick-end labeling (TUNEL)-labeled cells in the melatonin-treated group were prominently less compared with the MNU group. The apoptotic index (AI) of the melatonin treated group was significantly smaller compared with MNU group. **(D**, **E)** The mice in the 150 μg/kg group had larger retinal thickness and ONL thickness than those mice in the 50 and 100 μg/kg groups. **(F)** The apoptotic index (AI) of 150 μg/kg group was significantly smaller compared the 50 and 100 μg/kg groups (GCL, ganglion cell layer; IPL, inner plexiform layer; OPL, outer plexiform layer; ONL, outer nuclear layer; INL, inner nuclear layer; ANOVA analysis followed by Bonferroni's *post-hoc* analysis, ^#^*P <* 0.01, for differences between groups; *P < 0.01, for differences compared with previous dose group; n = 10).

The OCT examination showed that the retinal architecture of the MNU group was severely destroyed (**Figure 2A**). The retinal thickness in the MNU+vehicle group was not significantly different from that in the MNU group (*P* > 0.05; n = 10), suggesting that the surgical procedures of intravitreal injection would not affect the outcome of MNU induced retinal degeneration. Retinas of the MNU+melatonin group had relatively more intact architectures compared with MNU group. The retina thickness was significantly larger in the MNU+melatonin group than that in the MNU group (*P* < 0.01. n = 10). The ONL was undetectable in the histological sections of the MNU group, while several layers of cell nucleus were retained in the ONL of the MNU+melatonin group (**Figure 2B**). The mean ONL thickness of the MNU+melatonin group was significantly larger compared with the MNU group (*P* < 0.01; n = 10). Numerous TUNEL-positive cells were found in the ONL of the MNU group (**Figure 2C**). Conversely, less TUNEL-positive cells were found in ONL of the MNU+melatonin group. Apoptosis index (AI) was significantly smaller in the MNU+melatonin group than that in the MNU group (*P* < 0.01; n = 10). Furthermore, the dose-effect analysis showed that the mice in the 150 μg/kg group had larger retinal thickness (**Figure 2D**) and ONL thickness (**Figure 2E**) than those mice in the 50 and 100 μg/kg groups (*P* < 0.01; n = 10). On the other hand, the AI of 150 μg/kg group was significantly smaller compared the 50 and 100 μg/kg groups (*P* < 0.01; n = 10; **Figure 2F**). These morphological indicators in the 150 μg/kg group were not significantly different from those in the 200 and 250 μg/kg groups (*P* > 0.05; n = 10).

### Melatonin Mediated Protective Effects on Visual Function

**Figure 3:**
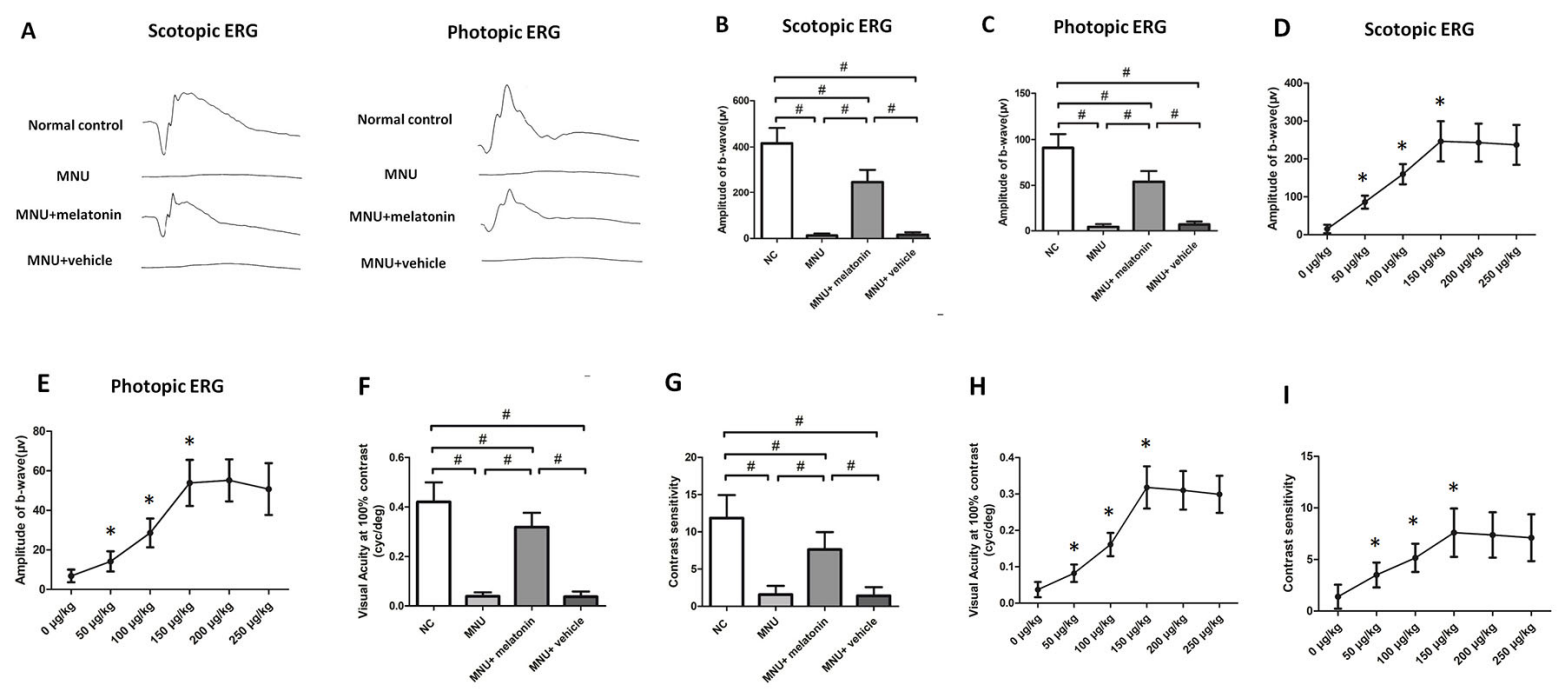
**(A)** The representative electroretinogram (ERG) waveforms of mice. There was a prominent reduction in the ERG amplitudes of the N-methyl-N-nitrosourea (MNU) group. The ERG waveforms in the MNU+ melatonin group were less deteriorated. **(B**, **C)** The scotopic and photopic b-wave amplitudes in the MNU+ melatonin group were significantly larger compared with the MNU group. **(D**, **E)** The mice in the 150 μg/kg group had larger b-wave amplitudes than those mice in the 50 and 100 μg/kg groups. The b-wave amplitudes in the 150 μg/kg group were not significantly different from those in the 200 and 250 μg/kg groups. **(F, G)** The mice in the MNU+vehicle group showed no significant improvement in optokinetic tests. Conversely, the visual acuity and contrast sensitivity were both significantly larger in the MNU+ melatonin group than those in the MNU group. The mice in the 150 μg/kg group had larger visual acuity **(H)** and contrast sensitivity **(I)** than those mice in the 50 and 100 μg/kg groups (ANOVA analysis followed by Bonferroni's *post-hoc* analysis, ^#^*P <* 0.01, for differences between groups; *P < 0.01, for differences compared with previous dose group; n = 10).

Typical ERG responses were induced in the normal controls (**Figure 3A**). In accordance with previous studies, the ERG responses of the MNU group were undetectable **[23]**. The b-wave amplitudes of the MNU+melatonin group were significantly larger compared with the MNU group (*P* < 0.01; n = 10; **Figures 3B, C**). The scotopic and photopic b-wave amplitudes in the MNU+melatonin group were 56.1 and 62.7% of the normal controls, respectively. These data suggested that melatonin therapy conferred pronounced protection on the visual function of MNU administered mice. The dose-effect analysis showed that mice in the 150 μg/kg group had larger b-wave amplitudes than those mice in the 50 and 100 μg/kg groups (*P* < 0.01; n = 10; **Figures 3D, E**). The b-wave amplitudes in the 150 μg/kg group were not significantly different from those in the 200 and 250 μg/kg groups (*P* > 0.05; n = 10). In the optokinetic tests, the mice of the MNU group were insensitive to the raster stimulus. The MNU+vehicle group had a visual acuity essentially identical to that in the MNU group (*P* > 0.05; n = 10; **Figure 3F**). The visual acuity in the MNU group was significantly smaller compared with the MNU+melatonin group (*P* < 0.01; n = 10). Moreover, the contrast sensitivity in the MNU+melatonin was significantly larger compared with the MNU group (*P* < 0.01; n = 10; **Figure 3G**). The dose-effect analysis showed that the mice in the 150 μg/kg group had larger visual acuity and contrast sensitivity than those mice in the 50 and 100 μg/kg groups (*P* < 0.01; n = 10; **Figures 3H, I**). Moreover, these functional indicators in the 150 μg/kg group were not significantly different from those in the 200 and 250 μg/kg groups (*P* > 0.05; n = 10).

### Melatonin Mediated Protective Effects on the Survival of Cone Photoreceptors

**Figure 4:**
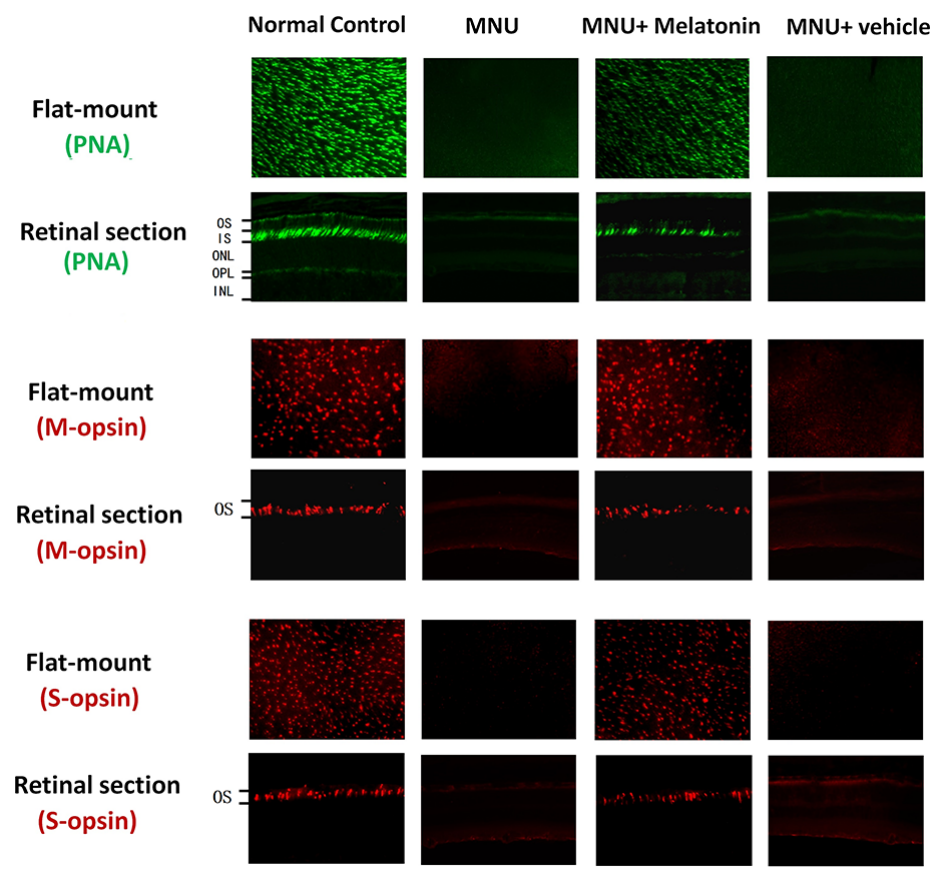
Immunostaining assay on the retinal sections and flat-mounts. The peanut agglutinin (PNA) fluorescence in the retinal specimens of N-methyl-N-nitrosourea (MNU) administered mice was extremely faint. Conversely, evident PNA fluorescence was found in the retinal specimens of the MNU+melatonin group. Moreover, the vitality of cone population was examined using opsin-specific antibodies. Both the M- and S-opsin positive cells were found in the retinal flat mount of MNU+melatonin group (OS, outer segments; IS, inner segments; OPL, outer plexiform layer; ONL, outer nuclear layer; INL, inner nuclear layer; ANOVA analysis followed by Bonferroni's *post-hoc* analysis).

Intense PNA fluorescence was found at the inner segments of the normal controls (**Figure 4**). The PNA fluorescence in the retinal sections of MNU group was extremely faint. Conversely, evident PNA fluorescence was found at the inner segments of the MNU+melatonin group. The retinal flat mounts of the MNU+melatonin group showed fairly well-preserved PNA fluorescence. The PNA-positive cell count averaged 723 ± 58 in MNU+melatonin group *versus* 15 ± 10 in the MNU group (*P* < 0.01; n = 10; **Table 1**). These findings suggested that the melatonin therapy result in a significant improvement in the cone photoreceptor survival. In the retinal flat mounts of MNU+melatonin group, the PNA-positive cell count of the dorsal-temporal (DT) quadrant was the largest, suggesting that the cone photoreceptors in this region were preferentially rescued by melatonin. Moreover, the M-opsin or S-opsin staining was undetectable in the retinal specimens of the MNU group. Conversely, these cone stainings were efficiently persevered in the retinas of the MNU+melatonin group. The average count of M- and S-opsin-positive cell was significantly larger in the MNU+melatonin group than that in the MNU group (*P* < 0.01; n = 10; **Table 1**). The average count of M- and S-opsin-positive cell in the MNU+melatonin group was 48.6 and 44.8% of the normal control, respectively.

**Table 1:** Cell counts in different quadrants of retina whole mounts.

Location	Normal control	MNU	MNU+Melatonin	MNNU+vehicle
PNA-Positive Cell Counts				
S T	1,698 ±113^‡§∮^	23 ±12^†§^	951±62^†‡∮^	20±13^†§^
IT	1,711 ±106^‡§∮^	18 ±9^†§^	754±55^†‡∮^	16±8^†§^
C	1,681 ±112^‡§∮^	10±5^†§^	660±51^†‡∮^	15±7^†§^
IN	1,662 ±114^‡§∮^	13±7^†§^	573±53^†‡∮^	19 ±8^†§^
SN	1,678±113^‡§∮^	9±6^†§^	699±60^†‡∮^	18±9^†§^
Average	1,686±112^‡§∮^	15±10^†§^	723±58^†‡∮^	18±11^†§^
M-Opsin Positive Cell Counts				
ST	1,240 ±71^‡§∮^	18+9^†§^	762±63^†‡∮^	19+7^†§^
IT	1,156±78^‡§∮^	13+9^†§^	595±51^†‡∮^	14+5^†§^
C	1,142 ±76^‡§∮^	9+5^†§^	429±40^†‡∮^	9+4^†§^
IN	1,140±69^‡§∮^	6+3^†§^	383±35^†‡∮^	8+3^†§^
SN	1,213±68^‡§∮^	8+5^†§^	698±57^†‡∮^	11+6^†§^
Average	1,178±73^‡§∮^	11±7^†§^	573±52^†‡∮^	12±5^†§^
S-Opsin Positive Cell Counts				
ST	305±48^‡§∮^	0^†§^	219±29^†‡∮^	0^†§^
IT	521 ±41^‡§∮^	0^†§^	246±35^†‡∮^	0^†§^
C	839±57^‡§∮^	8±6^†§^	320±38^†‡∮^	10±3^†§^
IN	920±66^‡§∮^	12±7^†§^	401±40^†‡∮^	19±6^†^
SN	805±60^‡§∮^	10±6^†§^	332±34^†‡∮^	15±5^†§^
Average	676±66^‡§∮^	6±5^†§^	303±37^†‡∮^	9±6^†§^

† *P* < 0.05 for difference compared with control group.

‡ *P* < 0.05 for difference compared with MNU group.

§ *P* < 0.05 for difference compared with MNU+Melatonin group.

∮ *P* < 0.05 for difference compared with MNNU+vehicle group.

All values are presented as mean ± SD; n = 10 per group.

### Melatonin Mediated Protective Effects on the Visual Signal Transmission

**Figure 5:**
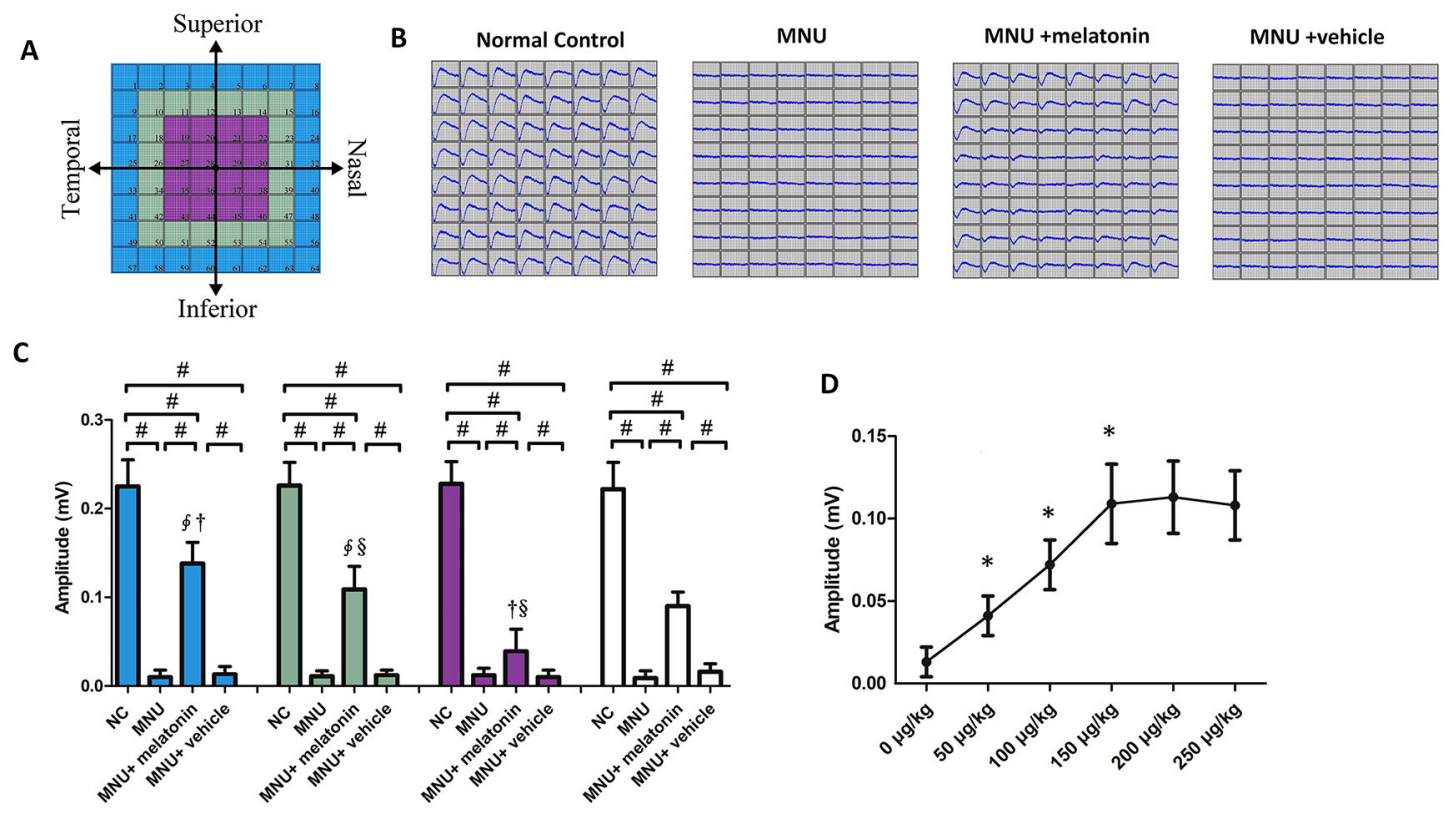
**(A)** Electrodes were classified into three categories according to their position. **(B)** The field potential waveforms were undetectable in the N-methyl-N-nitrosourea (MNU) group. The field potential waveforms of the MNU+melatonin group were effectively preserved. **(C)** The mean amplitude of field potential was significantly larger in the MNU+melatonin group than that in the MNU group. In the MNU+melatonin group, the field potentials in peripheral region had larger amplitudes than the other regions. **(D)** The mice in the 150 μg/kg group had larger amplitude of field potential than those mice in the 50 and 100 μg/kg groups. (ANOVA analysis followed by Bonferroni's *post-hoc* analysis, ^#^*P <* 0.01, for differences between groups; *P < 0.01, for differences compared with previous dose group; **^∮^***P <* 0.01, for differences compared with the central region; ^†^*P <* 0.01, for differences compared with the mid-peripheral region; ^§^*P <* 0.01, for differences compared with the peripheral region; n = 10).

**Figure 6:**
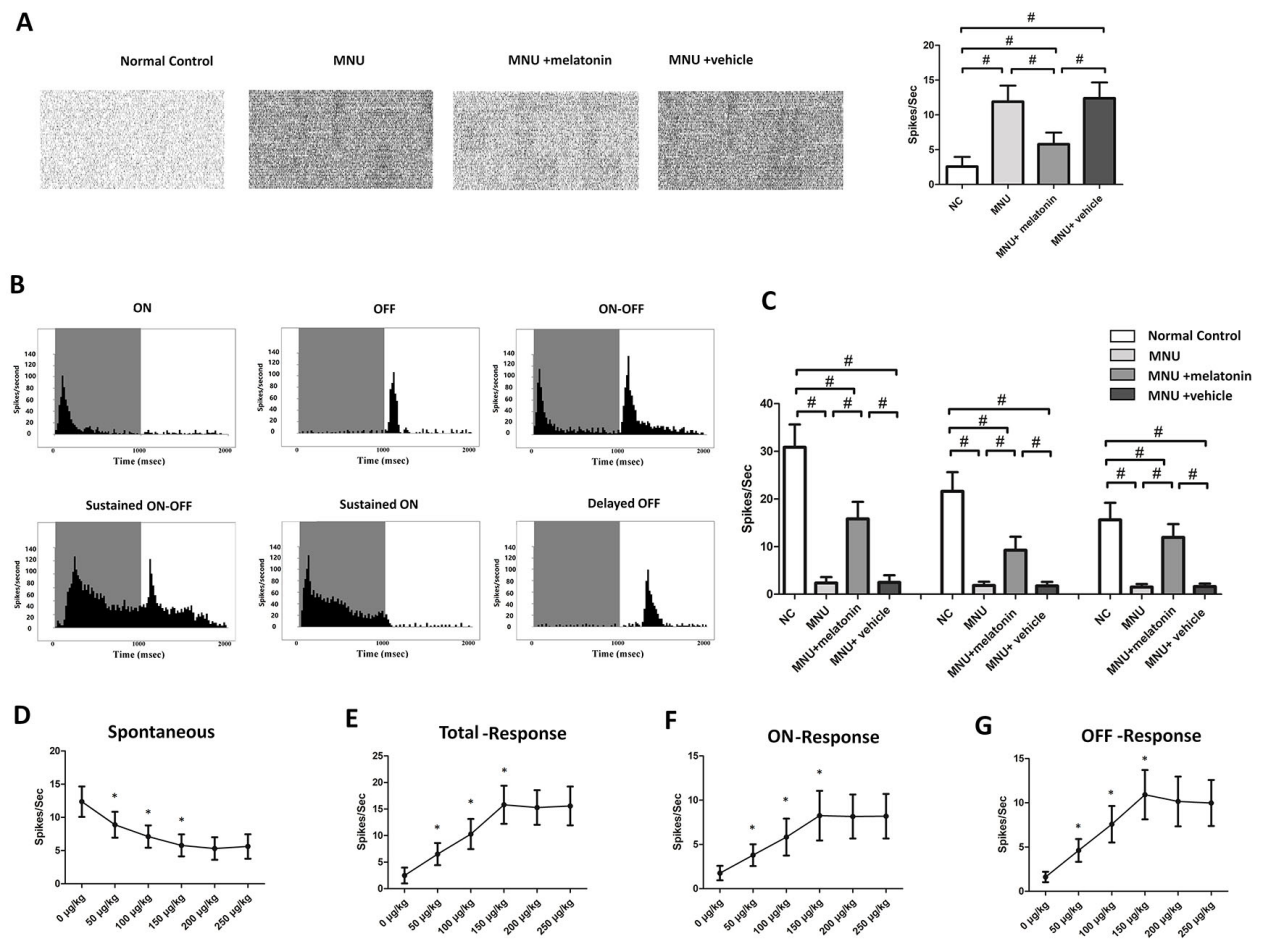
**(A)** The spontaneous firing spikes of retinal ganglion cells (RGCs). The spontaneous firing rate was significantly higher in the N-methyl-N-nitrosourea (MNU) group than that in the normal controls. The spontaneous firing rate was significantly lower in the MNU+melatonin group than that in the MNU group. **(B)** Main categories of RGCs were isolated on the basis of their peristimulus time histograms (PSTHs). **(C)** The total firing rate in the MNU group decreased significantly compared with normal controls. The total firing rate was significantly higher in the MNU+melatonin group than that in the MNU group. In the melatonin treated mice, the OFF pathway was more efficiently preserved than the ON pathway. The mice in the 150 μg/kg group had smaller spontaneous firing rate **(D)** and larger light induced firing rate **(E)** than those mice in the 50 and 100 μg/kg groups. The firing rates of ON and OFF response in the 150 μg/kg group were also significantly larger than those in the 50 and 100 μg/kg groups **(F, G)**. (ANOVA analysis followed by Bonferroni's *post-hoc* analysis, ^#^*P <* 0.01, for differences between groups; ^*^P < 0.01, for differences compared with previous dose group; n = 10).

MEA were classified into three categories: the central, the mid-peripheral, and the peripheral electrodes channels (**Figure 5A**). The field potential waveform was undetectable in the MNU group (**Figure 5B**). Conversely, the melatonin therapy preserved the field potential waveforms in the MNU+melatonin group. The mean amplitude of field potential was significantly larger in the MNU+melatonin group than that in the MNU group (*P* < 0.01; n = 10; **Figure 5C**). In MNU+melatonin group, the field potentials in the peripheral region had larger amplitudes than those in the mid-peripheral and central regions (*P* < 0.01; n = 10). The dose-effect analysis showed that the mice in the 150 μg/kg group had larger amplitude of field potential than those mice in the 50 and 100 μg/kg groups (*P* < 0.01; n = 10; **Figure 5D**). Moreover, amplitude of field potential in the 150 μg/kg group were not significantly different from those in the 200 and 250 μg/kg groups (*P* > 0.05; n = 10). The spontaneous firing rate was significantly higher in the MNU group than that in the normal controls (*P* < 0.01; n = 10) (**Figure 6A**). Melatonin therapy reduced significantly the spontaneous firing rate in degenerative retinas (*P* < 0.01; n = 10). Furthermore, the RGCs were categorized according to their light induced responses (**Figure 6B**). The total firing rate of light induced response was significantly higher in the MNU+melatonin group than that in the MNU group (*P* < 0.01; n = 10). Both the ON and OFF response intensities were significantly larger in the MNU+melatonin group than those mice in the MNU group (*P* < 0.01; n = 10). In particular, the OFF response was more efficiently preserved than the ON response. The ON response intensity was 38.1% of the normal controls, while the OFF response intensity was 69.9% of the normal controls. The dose-effect analysis showed that the mice in the 150 μg/kg group had smaller spontaneous firing rate and larger light induced firing rate than those mice in the 50 and 100 μg/kg groups (*P* < 0.01; n = 10; **Figures 6D, E**). The firing rates of ON and OFF responses in the 150 μg/kg group were also significantly larger than those in the 50 and 100 μg/kg groups (*P* < 0.01; n = 10; **Figures 6F, G**). These indicators in the 150 μg/kg group were not significantly different from those in the 200 and 250 μg/kg groups (*P* > 0.05; n = 10).

### Mechanisms Underlying Melatonin Induced Protective Effects

**Figure 7:**
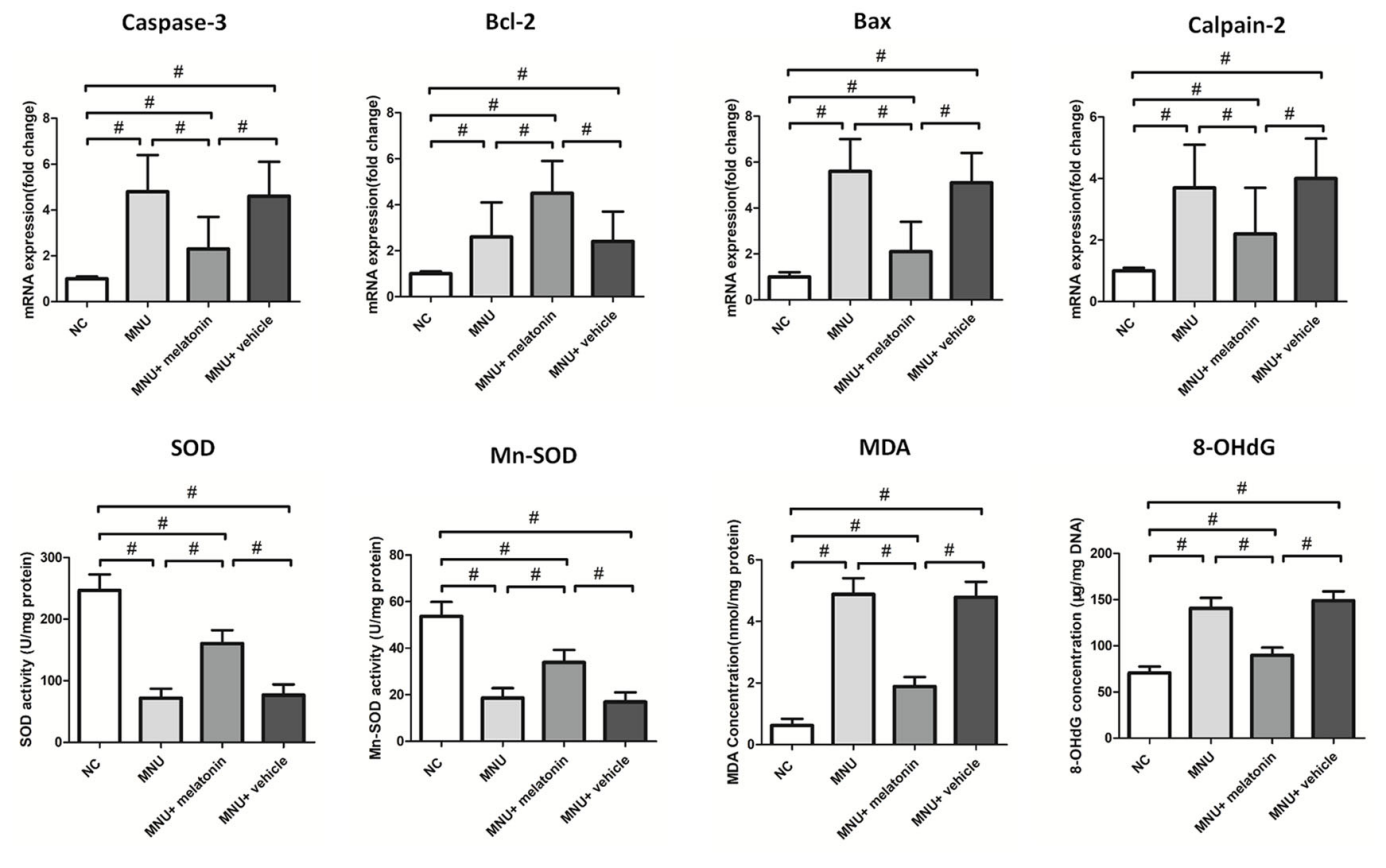
The messenger RNA (mRNA) levels of apoptotic factors in retina. The expression levels of malondialdehyde (MDA) and 8-hydroxy-2'-deoxyguanosine (8-OHdG) were significantly lower in the N-methyl-N-nitrosourea (MNU)+melatonin group compared with the MNU group. The retinal levels of superoxide dismutase (SOD) and manganese superoxide dismutase (MnSOD), were significantly higher in the MNU+melatonin group than those in the MNU group. (ANOVA analysis followed by Bonferroni's *post-hoc* analysis, ^*^P < 0.05, ^#^*P <* 0.01, for differences between groups; n = 10).

The messenger RNA (mRNA) levels of caspase-3, calpain-2, and Bax in the MNU+melatonin group were significantly lower compared with the MNU group (*P* < 0.01; n = 10; **Figure 7**). On the other hand, The mRNA level of Bcl-2 in the MNU+melatonin group was significantly higher compared with the MNU group (*P* < 0.05; n = 10). These findings suggested that the anti-apoptotic mechanism was, at least partly, responsible for the melatonin induced protection. The retinal MDA (a stable metabolite of lipid peroxidation) was 1.89± 0.303 nmol/mg in MNU+melatonin group *versus* 4.88± 0.521 nmol/mg in the MNU group (*P* < 0.01; n = 10). The retinal 8-OHdG (an indicator of DNA oxidative damage) was 89.71 ± 8.40 μg/mg in MNU+melatonin group *versus* 140.60 ± 11.37 μg/mg in the MNU group (*P* < 0.01; n = 10). These findings suggested that the melatonin therapy could alleviate the oxidative stress of degenerative retinas. Mitochondria impairments would result in the release of inter-membrane space proteins, and the subsequent activation of mitochondrial-dependent apoptosis (Mao and Sun, 2015). The retinal level of MnSOD, a mitochondrial protein with reactive oxygen species (ROS) scavenging potency, was 33.84 ± 5.37 U/mg in the MNU+melatonin group compared to 18.55 ± 4.29 U/mg for the MNU group (*P* < 0.01; n = 10), suggesting that melatonin therapy conferred beneficial effects on the mitochondria of photoreceptors. The retinal level of SOD was 160.455 ± 21.802 U/mg in the MNU+melatonin group compared to 71.831 ± 15.270 U/mg for the MNU group (*P* < 0.01; n = 10). These findings suggested that the melatonin therapy could enhance the activity of endogenous antioxidative enzymes in MNU administer mice.

## Discussion

Eye is a unique organ with transparent anterior structures and highly compartmentalized anatomy. These features will facilitate the drug delivery under direct visualization and subsequent non-invasive imaging *in vivo*. Intravitreal injection is a precise delivery approach which can maximize the drug intensity within eyeball (Haas et al., 2016). After injected into the vitreous humor, the therapeutic agents can touch a substantial fraction of the outer retina, avoiding the side effects on unwanted organs. To corroborate this point, we deliver melatonin into the vitreous cavity of the MNU administered mice. Melatonin could mitigate the photoreceptor loss in degenerative retinas. Melatonin is a lipophilic and hydrophilic molecule that can readily diffuse across concentration gradient, pass through the internal limiting membrane, and bathe the photoreceptor adequately (Doonan and Cotter, 2004; Huang et al., 2013). In this context, the intravitreal delivery would ensure high melatonin concentration in the retina. These findings lay the groundwork for future intraocular application of melatonin.

Gene therapy is a promising therapeutic strategy against RP (Fischer, 2016). However, the heterogeneous etiology of RP is challenging for any gene therapy that seek to rectify the primary defects. In the absence of genetic background for a given RP patient, pharmacologic therapy could be a temporizing measure until a genetic diagnosis can be made and a specific therapy devised (Drack et al., 2012). The common pathological process underlying photoreceptor degeneration might afford an alternative therapeutic target (Sancho-Pelluz et al., 2008). For instance, excessive oxidative stress contributes to the photoreceptor degeneration with variable etiologic backgrounds (Tsuruma et al., 2012; Narayan et al., 2016; Donato et al., 2019). This is the rationality to build the therapeutic strategy on antioxidants. Melatonin is a circadian hormone that is primarily produced by the pineal gland (Back et al., 2016; Reiter et al., 2016). Photoreceptors are also capable of synthesizing melatonin and releasing them into retinal tissue (Tosini et al., 2012). Retinal melatonin participates in a broad spectrum of physiological activities, such as maintaining the light responsiveness, regulating intraocular pressure, and adjusting circadian rhythms (McMahon et al., 2014; Crooke et al., 2017). A recent study shows that endogenous melatonin can modulate photoreceptor viability *via* the MT1 receptors (Baba et al., 2009). On the other hand, systematic administration of exogenous melatonin is reported to protect the RGCs from hypoxic injuries (Kaur et al., 2013). The exogenously applied melatonin can stabilize the mitochondria and reduces the cytochrome c leakage into the cytosol of RGCs. Another *in vitro* study shows that adding the melatonin to culture solution can protect photoreceptors from light-induced oxidation (Marchiafava and Longoni, 1999). For P23H rats carrying a mutation defect, adding melatonin into the drinking water alleviates the photoreceptors loss and visual impairments (Lax et al., 2011). Daily injection of melatonin also retards the photoreceptor degeneration in the rd10 and rds mice (Liang et al., 2001; Xu et al., 2017). More excitingly, a clinical investigation shows that the age-related macular degeneration (AMD) patients have lower melatonin level, and the oral melatonin supplements delay the macular degeneration and improve the visual acuity of these patients (Crooke et al., 2017). Herein, we show that a single intravitreal injection of melatonin is potent enough to alleviate the MNU induced photoreceptor degeneration. Typically, the MNU induced photoreceptor degeneration accomplishes within 1 week (Tsubura et al., 2011; Tsuruma et al., 2012). In this context, robust protective strategies are necessary to arrest the rapid photoreceptor apoptosis. In particular, the melatonin mediated protection follows a dose-dependent manner. As a well known anti-oxidant, melatonin is not only able to scavenge directly the free radical, but also to enhance the production of endogenous anti-oxidative enzymes (Rodriguez et al., 2004). We show that the melatonin therapy enhances the expression levels of Cu-Zn-SOD and MnSOD, both of which are ubiquitous oxidation protectors in retina (Akpinar et al., 2007; Biswal et al., 2017). On the other hand, melatonin could reduce the level of MDA and 8-OHdG, which are classic markers of lipid and DNA peroxidation (Celebi et al., 2002; Deliyanti et al., 2018). These findings suggest that melatonin may be beneficial for the retinopathies related to oxidative stress. Nevertheless, several pharmacological issues should be addressed before further clinical application. Melatonin must be applied at the appropriate chance and in the feasible way to RP patients. The optimal dosages, administration routes, and therapeutic time window of melatonin therapy should be well characterized. Additionally, the potential adverse effects of the high-dose melatonin should be evaluated by a large scale clinical trial.

Photoreceptor apoptosis reduces the oxygen consumption and exacerbates the oxidative stress in retinal tissue (Campochiaro and Mir, 2018). Oxidative stress in turn activates the apoptotic cascade, and accelerates the death of photoreceptors (Talcott et al., 2011). TUNEL assay is a reliable method to identify the apoptotic cells in retina (Nagar et al., 2017). Our TUNEL results show that intravitreal injection of melatonin is able to inhibit the MNU induced photoreceptor apoptosis. Furthermore, melatonin reduces significantly the mRNA level of apoptotic factors. These findings suggest that that modulating the apoptotic threshold might be beneficial for photoreceptor survival (León et al., 2005; Fernández et al., 2015; Yang et al., 2015). Accordingly, melatonin might provide a mutation-independent medication that can be generalized to RP patients with different etiologic backgrounds.

Our MEA data shows that the photoreceptors in peripheral retina are more efficiently preserved than other areas. As a metabolically active tissue, retina is characterized by the intense oxygen consumption (Yu and Cringle, 2001). The blood supply of central retina depends exclusively on the choroidal vessel system, while the peripheral retina relies on both the retinal and choroidal vessel system (Blasiak et al., 2016). Hence, the detrimental factors in the peripheral regions can be eliminated instantly by blood circulation. Previous studies have shown that the photoreceptors in the central retina are more vulnerable to chemical or pathogenetic factors than those in the peripheral region (Jimenez et al., 1996; Stone et al., 1999; Homma et al., 2009). For instance, the MNU induced photoreceptor degeneration is remarkably more severe in the central retina, while the peripheral photoreceptors can survive longer as their blood supply is much more abundant. Therefore, the differences in therapeutic efficiency should be ascribed to the comparative vulnerability across retinal regions (Tao et al., 2015a; Tao et al., 2015b).

Similar to other RP animal models (Masland, 2001), spontaneous RGCs hyperactivity occurs in the MNU administered mice. The spontaneous RGCs hyperactivity is detrimental to the visual signaling, since it would add unnecessary noise into retinal circuits (Marc et al., 2007; Lin et al., 2009; Wu, 2009; Bisti, 2010; Barrett et al., 2015). Melatonin therapy can restrain the spontaneous hyperactivity and enhance the light induced response in MNU administered mice. Exogenous melatonin is unlikely to alter the cellular membrane, dominant receptive fields, and intrinsic activities of the RGCs, since the melatonin receptors are rarely expressed on the RGCs of mice (Wiechmann and Sherry, 2013). A possible mechanism underlying these benefits may be attributed to the melatonin induced effects on the retinal circuits which are presynaptic to the RGCs (Baba et al., 2009). RGCs receive simultaneously the excitatory inputs from bipolar cells and the inhibitory glycinergic inputs from amacrine cells. Electrophysiological activity of RGCs is shaped delicately by this antagonistic system. Any changes in the presynaptic inputs would produce secondary effects on the RGCs (Protti et al., 1997; Euler and Schubert, 2015). It is noteworthy that melatonin plays a critical role in the signaling of amacrine cells (Lundmark et al., 2006; Huang et al., 2013). Melatonin can potentiate the glycine receptor-mediated post-synaptic currents in RGCs, thereby activating the inhibitory inputs from glycinergic amacrine cells (Zhao et al., 2010). Therefore, it is reasonable to speculate that the melatonin inhibits the spontaneous RGCs hyperactivity *via* the glycinergic system. Moreover, visual signal pathway reorganization occurs in the melatonin treated mice: the balance between ON and OFF pathway is disturbed, and the OFF signal pathway would dominate the visual signal transmission. Glycinergic amacrine cells are essentially involved in the crossover inhibition between ON and OFF pathways in the inner retinal circuits (Hsueh et al., 2008; Molnar et al., 2009). By modulating the inhibitory signals from glycinergic amacrine cells, melatonin may drive the OFF-RGCs to a more hyperpolarized level, which would enable these cells to detect subtle contrast at night (Zhao et al., 2010; Wiechmann and Sherry, 2013). These benefits may collectively contribute to the improved efficiency of visual signaling (Lin et al., 2009; Jones et al., 2016).

Admittedly, some shortcomings are implied in this study. While the melatonin treatment is able to ameliorate the MNU induced photoreceptor degeneration, these findings confine themselves to study on animal models. MNU is equally toxic to both rod and cone photoreceptors. The observation that cones are also killed by MNU toxicity does not correspond to cone degeneration in RP patients, since demise of this population occurs in a secondary wave of cell death, long after rods have degenerated (Narayan et al., 2016). Furthermore, the rodent retinal has a cone distribution primarily as a ring in the equatorial retina which is quite different from the fovea dependence on cones in human (Szél et al., 1996). The two cone populations (M- and S-cone) are not uniformly distributed across the mouse retina. Our immunostaining results showed that M-cones in DT quadrant are preferentially rescued by melatonin treatment. This disequilibrium is also found in the hereditary RP animal models: the superior quadrant with patches of late-surviving cones is the most resistant in the rd1 mouse (Narayan et al., 2016). However, regarding the situation in RP patients, it is still disputed if an isolated protection of cones would work once the rods are lost. Therefore, the melatonin induced beneficial effects on cones should be further validated in the large animal model which has retinal architectures more similar to human.

In conclusion, intravitreal delivery of melatonin can alleviate the MNU induced photoreceptor degeneration. Melatonin can also rectify the abnormities in visual signal transmission within inner retinal circuits. Melatonin affords these benefits by inhibiting apoptosis and mitigating oxidative stress. These findings highlight the possibility that intravitreal delivery of melatonin might be beneficial for the visual function of RP. Further studies are necessary to characterize the exact mechanism underlying the melatonin induced protection.

## Data Availability Statement

All datasets generated for this study are included in the article/supplementary material.

## Ethics Statement

The animal study was reviewed and approved by Chinese PLA General Hospital.

## Author Contributions

CL, YiT, AY: performed the experiments, analyzed the data. YeT, CL, JZ: drafted the manuscript. CL, XZ, JZ: acquired the data and provided material support. YeT, CL, JZ, XZ: analyzed and interpreted the data, revised the manuscript and finally approved the version of the manuscript for publication. CL, AY, XZ, JZ, YiT: contributed to the conception and design of the study, analyzed and interpreted the data, supervised the study, provided the project funding.

## Funding

This study is supported in part by the National Natural Science Foundation of China [No. 81600767]; the Shaanxi Province Innovation Capacity Support Program (2019KJXX-090); the Manned Space Advance Research Program (020103); the Open Foundation of National Key Laboratory for Renal Disease in Chinese PLA General Hospital [No. KF-01-114].

## Conflict of Interest

The authors declare that the research was conducted in the absence of any commercial or financial relationships that could be construed as a potential conflict of interest.
